# From dialogue to action: Advancing diabetes prevention and care in South Africa

**DOI:** 10.4102/phcfm.v18i2.5590

**Published:** 2026-09-30

**Authors:** Patrick Ngassa Piotie, Melanie Pienaar

**Affiliations:** 1 School of Health Systems and Public Health, Faculty of Health Sciences, University of Pretoria, Pretoria, South Africa; 2 Department of Public Health, Medical School, Nelson Mandela University, Gqeberha, South Africa; 3 Diabetes Alliance South Africa, Pretoria, South Africa; 4 School of Nursing, Faculty of Health Sciences, University of the Free State, Bloemfontein, South Africa

South Africa is navigating a demographic and epidemiological transition that threatens to overwhelm its public health infrastructure.^1^ Diabetes mellitus is a leading cause of death and disability.^2^ However, the national response remains disproportionately modest compared to the scale of this threat. The rising prevalence, late diagnoses, suboptimal management and increasing complications continue to place pressure on already strained primary care services.^3^

The economic burden is equally concerning, with projections suggesting that diabetes-related costs could reach R35 billion annually by 2030,^4^ positioning the condition not only as a clinical priority but also as a significant economic and developmental risk to the nation’s pursuit of Sustainable Development Goal 3. These challenges are further compounded by persistent inequities in access to care, particularly in underserved communities, and by broader social determinants of health such as poverty, food insecurity and urbanisation that shape diabetes risk and outcomes.

While South Africa has a commendable history of tackling infectious diseases through massive, coordinated state action, the response to non-communicable diseases (NCDs), and diabetes specifically, remains fragmented. Despite the existence of clinical guidelines, the reality for most South Africans remains one of late diagnosis, erratic medicine supply and suboptimal glycaemic control.^5^

Against this backdrop, the *2025 Diabetes Summit*, themed ‘Innovate for Impact: Transforming the Future of Diabetes in South Africa’, marked a critical inflection point. The Summit brought together policymakers, healthcare providers, researchers, civil society organisations and people living with diabetes, and was grounded in a whole-of-society approach. Through plenary sessions, technical workshops and stakeholder dialogues, the Summit provided a space to reflect on persistent challenges and identify practical, contextually grounded solutions capable of driving impact.

This Special Collection serves as an academic distillation of the Summit’s deliberations and provides a rigorous multidimensional analysis of the current landscape of diabetes prevention and care in South Africa. The contributions span diverse yet interconnected themes, ranging from the strengthening of diabetes education and peer support to multisectoral action for NCDs and mental health. The collection underscores the centrality of lived experience and advocacy while exploring the role of innovation in transforming service delivery. Crucially, these articles bridge the gap between high-tech aspirations such as the equitable roll-out of continuous glucose monitoring (CGM) and the grounded realities of managing diabetes in underserved rural communities.

Several cross-cutting priorities emerge from this body of work. Firstly, there is a clear need to strengthen integrated chronic care models within primary care, building on lessons and infrastructure from human immunodeficiency virus (HIV) and tuberculosis (TB) programmes to improve screening, continuity of care and community-based support for people living with diabetes. Secondly, differentiated models of care are essential: facility-based services alone cannot meet the long-term needs of people with diabetes, and stronger linkages with community-based and peer-led interventions are critical for sustained engagement and self-management. Thirdly, innovation must be pursued through an equity lens. Technological advances, including digital tools and CGM, hold promise but risk widening disparities if access is not deliberately addressed. Fourthly, the persistent gap between policy and implementation requires stronger accountability mechanisms, improved coordination across levels of government and sustained political commitment. Fifthly, anchoring care design in the lived experiences of people with diabetes is essential for developing responsive and effective care models. Finally, diabetes prevention and control require multisectoral action, extending beyond the health sector to include coordinated efforts across finance, education, agriculture, trade, social development and urban planning.

These priorities carry important implications for national policy. South Africa’s National Strategic Plan for the Prevention and Control of NCDs sets ambitious targets for diagnosis, treatment and control. However, achieving these targets will require more than incremental progress. The complexity of the challenges highlighted in this Special Collection underscores the limitations of fragmented responses and points to the need for a more cohesive and strategic national approach. In this context, the development of a dedicated *National Diabetes Strategy*, adequately resourced, coordinated and multisectoral, may offer a pathway to strengthen implementation, improve accountability and enable the scaling of effective interventions.

The most significant output of the 2025 Summit, however, was not a research article but the *Johannesburg Declaration for Accelerated Action on Diabetes*. This Declaration represents a unified demand for the South African government to elevate diabetes to the status of a national public health emergency. It articulates a vision where the urgency of the ‘90-90-90’ targets used for HIV is applied with equal vigour to diabetes: 90% of people screened, 90% of those diagnosed receiving treatment, and 90% of those on treatment achieving controlled blood glucose levels.^6^

This Special Collection is a repository of evidence intended to catalyse action and fuel a movement. The transition from dialogue to action is not a choice; it is a necessity. If we fail to translate these insights into a scalable, coordinated national response, the ‘silent killer’ will continue to erode South Africa’s health and prosperity. The opportunity now is to ensure that the knowledge generated is translated into practice and that the commitments articulated are matched by sustained, coordinated, and accountable action.

## References

[1] Agyemang C, Tetteh J, Mbaye MN, et al. Burden and determinants of diabetes in sub-Saharan Africa. Lancet Diabetes Endocrinol. 2026;14(6):498–511. 10.1016/S2213-8587(26)00065-3 42044651

[2] Statistics South Africa. Mortality and causes of death in South Africa: Findings from death notification, 2022. Statistical Release. Report No.: P0309.3. Pretoria: Statistics South Africa; 2025.

[3] Ikhile D, Seidu S, Omodara D, Katte JC, Ramaiya K, Khunti K. Diabetes-related complications and multiple long-term conditions in sub-Saharan Africa: Determinants and management strategies. Lancet Diabetes Endocrinol. 2026;14(6):512–522. 10.1016/S2213-8587(26)00036-7 42044653

[4] Erzse A, Stacey N, Chola L, Tugendhaft A, Freeman M, Hofman K. The direct medical cost of type 2 diabetes mellitus in South Africa: A cost of illness study. Glob Health Action. 2019;12(1):1636611. 10.1080/16549716.2019.1636611 PMC7012049 31282315

[5] Ngassa Piotie P, Webb EM, Rheeder P. Suboptimal control and failure to intensify therapy for South Africans with type 2 diabetes: An audit of diabetes management at primary health care facilities. J Endocrinol Metab Diabetes S Afr. 2024;29(1):37–42. 10.1080/16089677.2024.2311497

[6] Marinda E, Simbayi L, Zuma K, et al. Towards achieving the 90–90–90 HIV targets: Results from the South African 2017 National HIV Survey. BMC Public Health. 2020;20(1):1375. 10.1186/s12889-020-09457-z PMC7487872 32907565

